# Comparison of Semi-automated Late Gadolinium Enhancement Quantification Techniques in Measuring Septal Fibrosis in Patients with Dilated Non-Ischemic Cardiomyopathy

**DOI:** 10.1186/1532-429X-18-S1-P287

**Published:** 2016-01-27

**Authors:** Yoko Mikami, Aidan K Cornhill, Naeem Merchant, Carmen P Lydell, Andrew G Howarth, James A White

**Affiliations:** 1 Stephenson Cardiac Imaging Centre, Libin Cardiovascular Institute of Alberta, University of Calgary, Calgary, AB Canada; 2 Diagnostic Imaging, University of Calgary, Calgary, AB Canada; 3 Cardiac Sciences, University of Calgary, Calgary, AB Canada

## Background

Mid-wall striae pattern late gadolinium enhancement (LGE) has been shown to independently predict future outcomes in patients with dilated non-ischemic cardiomyopathy (NICM). Standardized quantification tools are of particular interest for clinical translation of this novel imaging marker. However, signal intensity (SI) based threshold techniques, such as Signal Threshold versus Reference Mean (STRM) and Full Width at Half Maximum (FWHM), have been inconsistently applied and their performance in this population is unknown. The purpose of this study was to compare the accuracy of semi-automated LGE quantification techniques against expert-prescribed threshold assignment in patients with NICM.

## Methods

LGE images acquired using a 3.0T MRI system were assessed. Nineteen patients with NICM (14 male, mean age 58 years) and visually confirmed mid-wall septal LGE were studied. TI was manually adjusted to obtain optimal myocardial nulling (range: 280 to 340 ms). Total LGE mass in the septal region (defined as the region between and inclusive of RV insertion points) was quantified using 5 semi-automated techniques (STRM thresholds of ≥2,≥3,≥5 SD above mean SI of reference remote myocardium, FWHM, and Peak of Remote Tissue (PRT) threshold techniques). All analysis was performed using commercially available software (cvi^42^, Circle Cardiovascular Inc.). Reference tissue regions of interest (ROI) were selected with careful attention to avoid artifact or blood pool. Peak SI in the remote tissue was automatically detected within remote tissue ROI by the software. Total septal LGE mass was compared to blinded expert segmentation of the same region using manual adjustment of the SI threshold. Bland-Altman analysis and intra-class correlation coefficients (ICC) were then applied.

## Results

**Figure 1:**
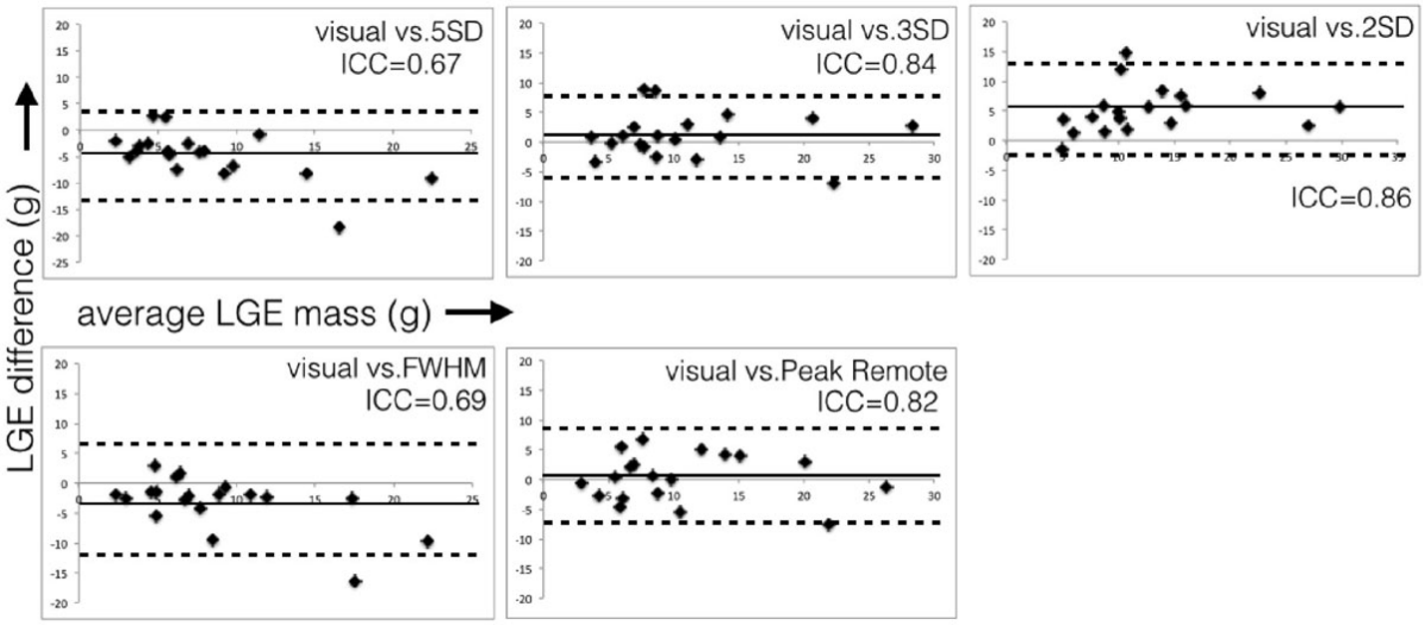
**Bland-Altman plots between each semi-automate technique and manual expert segmentation**.

Overall, the STRM ≥3SD and PRT techniques showed greatest agreement with expert manual segmentation for the quantification of septal LGE (STRM ≥3SD: ICC = 0.84, mean difference and 95% limits of agreement = 1.12 ± 7.56 g), (PRT: ICC = 0.82, mean difference and 95% limits of agreement = 0.32 ± 7.84 g). STRM ≥ 5SD and FWHM techniques systematically underestimated septal LGE mass, and STRM ≥ 2SD technique over-estimated septal LGE mass (Figure 1).

## Conclusions

STRM ≥ 3SD and PRT segmentation best approximate expert-defined septal LGE extent in patients with NICM. FWHM segmentation systematically underestimates and STRM ≥2SD overestimates septal LGE mass in this patient population.

